# IGF-II-secreting bladder tumour presenting as a fall in a 91-year-old

**DOI:** 10.1530/EDM-25-0090

**Published:** 2025-12-02

**Authors:** Imaan Hirji, Shwetha Sairam, Hiro Khoshnaw

**Affiliations:** Royal Surrey NHS Foundation Trust, Guildford, United Kingdom

**Keywords:** rare diseases/syndromes, growth factors, ageing, endocrine cancers

## Abstract

**Summary:**

Insulin-like growth factor II (IGF-II)-secreting tumours represent a subset of non-islet cell tumours and are a rare but potentially life-threatening cause of paraneoplastic hypoglycaemia. They are predominantly associated with epithelial or mesenchymal tumours but have also been reported in some hepatocellular carcinomas or retroperitoneal sarcomas. Definitive treatment involves surgical resection of the mass; however, if that is not possible, then various forms of medical management can be trialled. This paper presents the case of a 91-year-old woman admitted to hospital with worsening pelvic pain. Throughout the admission, she was found to have recurrent episodes of hypoglycaemia that, when investigated, were attributed to a bladder tumour. She was diagnosed with an IGF-II-secreting non-islet cell tumour based on imaging, biochemistry, and clinical judgement. Due to her age and limited physical reserve, she declined further investigation or surgical management, leading to trial and successful medical management of her condition. This case highlights a rare presentation of IGF-II-mediated non-islet cell tumour hypoglycaemia arising from the bladder in an elderly patient, which has minimal representation in the literature.

**Learning points:**

- Unexplained and recurrent episodes of hypoglycaemia should raise clinical suspicion of a paraneoplastic process that requires further investigation.
- It is important to maintain a broad range of differentials when approaching a frail patient with recurrent hypoglycaemia. Management should be guided by discussion with a multidisciplinary team and patient preference.
- IGF-II-secreting tumours are widely associated with mesenchymal tumours; however, this case presents a rare possible association with bladder tumours.
- Surgical resection of NICTH tumours is the mainstay of treatment, but when it is not feasible, other medical management should be explored.

## Background

Non-islet cell-secreting tumours are rare but highly dangerous paraneoplastic processes that are often associated with tumours of the epithelium or mesenchyme (1). They are usually mediated by insulin-like growth factor (IGF)-II and cause episodes of profound hypoglycaemia. IGF-II is a bioactive polypeptide that causes insulin-like activity by binding to insulin and IGF receptors (1). Patients present with fasting hypoglycaemia caused by suppression of endogenous insulin, ketones, growth hormone, and insulin receptors. It is mainly treated with intravenous dextrose therapy and supportive measures; however, symptoms often reappear only a few hours later (1).

This case is of significant interest to clinicians due to its rare presentation of non-islet cell tumour hypoglycaemia (NICTH) secondary to a bladder tumour, a previously undocumented association. This article presents a case report of a non-islet cell-secreting tumour in an elderly patient. IGF-II-secreting tumours are uncommon, and their occurrence in the bladder has not been well described in the literature, making this a novel and unusual presentation. This case also highlights the diagnostic challenges of investigating hypoglycaemia in elderly patients, where symptoms are often mistakenly attributed to frailty or age-related decline.

## Case presentation

A 91-year-old female presented to hospital in July 2023 with reduced mobility and worsening pelvic pain. She was noted to be a relatively well 91-year-old with a clinical frailty score (CFS) of 5. She had a functional baseline of being independently mobile, was able to walk up and down stairs, and could manage all her activities of daily living without support.

**Figure 1:**
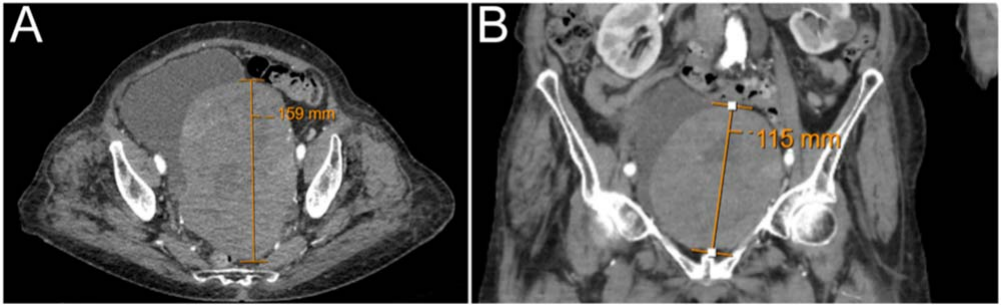
CT thorax, abdomen, and pelvis with contrast. Mass seen in the (A) axial view and (B) coronal view.

When presenting to the hospital in July, she was significantly off her baseline. She was unable to mobilise without assistance and could barely weight bear due to the severity of the pain. Given the recent functional decline and concerns regarding a possible unwitnessed fall, an urgent CT scan of the pelvis was performed. This scan revealed a significantly enlarged left-sided pelvic mass compared to prior imaging from 2021. The mass appeared to arise from the bladder with extrinsic compression of adjacent structures (Fig. 1). Differential diagnoses included bladder or ovarian malignancy.

## Investigation

**Figure 2:**
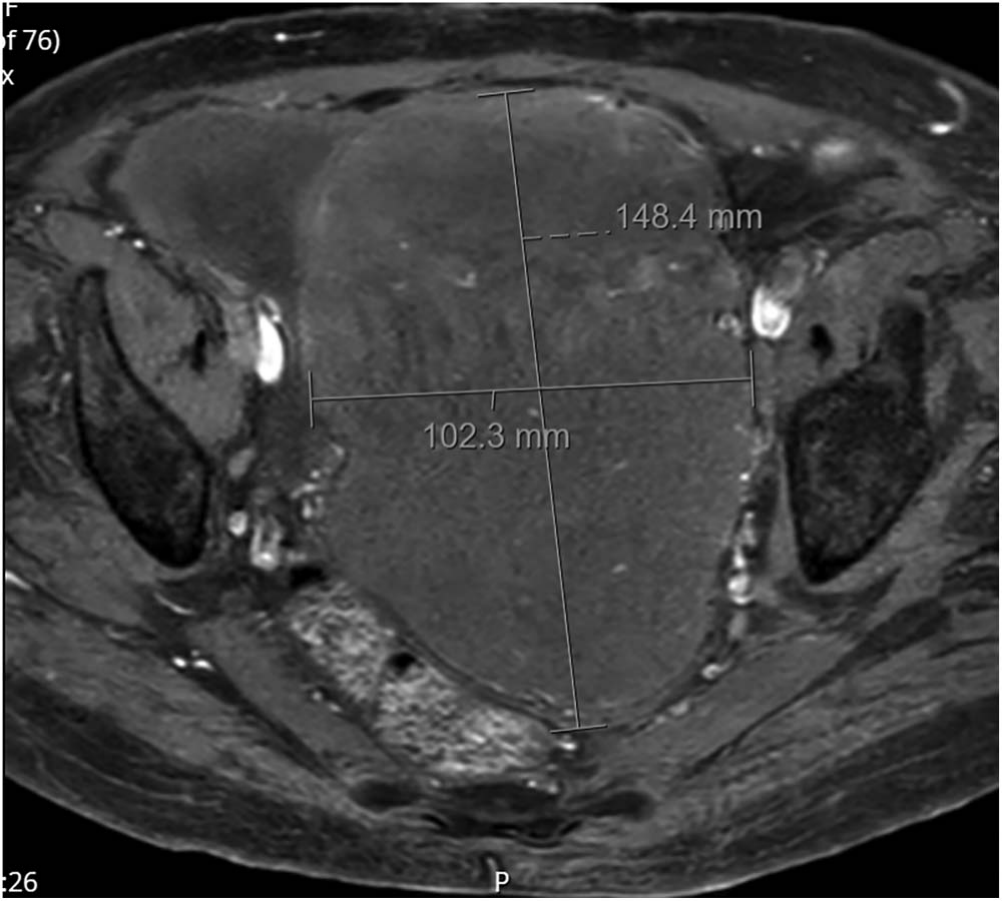
MRI pelvis axial view.

Further imaging with MRI pelvis confirmed a 15.2 × 11.3 × 11.8 cm well-defined intravesical mass with mixed signal characteristics and diffusion restriction (Fig. 2). The radiological appearance was consistent with a bladder leiomyoma. There was no evidence of extravesical extension or pelvic lymphadenopathy. On further history taking, the patient reported a surgical history including a hysterectomy and salpingo-oophorectomy in 1993 for chronic cervicitis, a fully resected leg melanoma in 1999, and a bladder tumour resected in 2000, reported by the patient as a sarcoma. These investigations and treatments were done in Spain; therefore, no histopathological records were available for review.

Throughout the first few days of her admission, the patient experienced multiple profound episodes of hypoglycaemia. Her blood sugar levels dropped to 1.9 mmol/L, and she was very symptomatic with these readings. She fulfilled Whipple’s triad: symptoms of hypoglycaemia, a low blood glucose level, and relief of symptoms after raising blood glucose levels, and therefore was referred to the endocrinology team.

Endocrine evaluation suggested many investigations with the impression of non-islet cell tumour hypoglycaemia (NICTH). During the next episode of hypoglycaemia, bloods were taken. Biochemistry during a hypoglycaemic episode revealed the results preseted in Table 1.

**Table 1:** Biochemistry results during an episode of hypoglycaemia.

	Reference range	Reported result	Interpretation
Insulin, μIU/mL	2–25	<10	Normal-low
C-peptide, pmol/L	298–2,350	<94	Low
GH, ng/mL	Random: <10; fasting: <5	0.6	Low
IGF-1, ng/mL	100–300	324.1	High
IGF-2, nmol/L	18–38	49.1	High
BHB, μmol/L	Fasting: <250	132	
Cortisol, nmol/L	140–690	329	
TSH, mIU/L	0.4–4.0	0.75	
Free T4, pmol/L	9–19	13.1	
Free T3, nmol/L	3.1–6.8	3.7	

GH, growth hormone; TSH, thyroid-stimulating hormone; BHB, beta-hydroxybutyrate.

Low-normal insulin levels and low C-peptide levels suggest suppression of endogenous insulin production. Within the context of hypoglycaemia, this pattern indicates that the hypoglycaemia is not caused by insulin overproduction or exogenous insulin administration but rather by an insulin-like substance. Biochemistry demonstrated suppressed insulin, C-peptide, and GH, with a disproportionately elevated IGF-II level and a raised IGF-II to IGF-I ratio, consistent with non-islet cell tumour hypoglycaemia.

During this time, the patient had been referred to the Urology Multidisciplinary Team (MDT) meeting. Imaging and discussion confirmed that the bladder lesion had characteristics consistent with a leiomyoma. Recommendation was made for whole body staging CT, MRI, and tissue biopsy.

## Treatment

After gathering information from all relevant teams, a diagnosis of non-islet cell tumour hypoglycaemia was made, likely secondary to the bladder tumour. These findings were presented to the patient and her family. After discussion, the patient declined further investigations and opted for best supportive care. Due to the likely paraneoplastic cause of hypoglycaemia, prednisolone 30 mg daily was initiated with symptom improvement.

## Outcome and follow-up

The patient was discharged on a tapering steroid regimen and eventually maintained on prednisolone 10 mg daily. She was then symptomatic again on this lowered dose, causing the dose to be increased to 30 mg per day. Over the next year, the patient became increasingly frail and was admitted to hospital four more times with various aetiologies. Recurrent hypoglycaemia did not contribute to further admissions to hospital, and she maintained adequate symptom control on 30 mg of prednisolone. After some time, the patient and her family chose to focus on comfort and keeping her at home. She was discharged with a Proactive Elderly Advance CarE (PACE) document and with hospital-at-home services, community palliative care, and GP follow-up. She passed away at home about 1 year later.

## Discussion

### Diagnostic difficulties

In this case of a 91-year-old frail woman with multiple comorbidities and recent trauma, establishing a definitive diagnosis was difficult. Her initial symptoms – recurrent unwitnessed falls and progressive functional decline – were initially attributed to age-related frailty and recent injury. During an investigation for possible hip fractures, the pelvic mass was found incidentally. This piece of information was vital in explaining many other symptoms that could have easily been overlooked. This case highlights the importance of maintaining a broad differential diagnosis in older patients, particularly when symptoms are vague, multifactorial, or potentially attributable to age-related factors or existing comorbidities.

Another difficulty in this case was the patient’s refusal to undergo further investigation. As no histopathology was available, the diagnosis was made based on MRI findings and clinical judgement. Later, the patient disclosed a prior resection of a bladder sarcoma in Spain, though no records were available, and the history was based on recollection. This raised the possibility that the mass could have been a bladder leiomyosarcoma – a more aggressive and better-documented case in the literature – attributing to the underlying cause of the patient’s symptoms.

### Bladder leiomyomas and leiomyosarcoma

An important limitation of this report is the absence of histopathological confirmation, as the patient declined a biopsy. Although imaging and biochemical evidence strongly support NICTH, the precise tumour type (leiomyoma vs leiomyosarcoma) remains speculative.

Bladder leiomyomas are a rare form of mesenchymal neoplasms, accounting for less than 0.5% of all bladder tumours (2). Diagnosis can be made through various investigations, including ultrasound (US), computed tomography (CT) scans, magnetic resonance imaging (MRI), cystoscopy, and histopathology (2). On imaging, it can be challenging to differentiate between leiomyomas and leiomyosarcomas. Both lesions appear as large, polypoid masses near the dome of the bladder and show low signal intensity on T2-weighted MRI (3). Leiomyosarcomas often demonstrate invasion into local structures, poorly defined borders, and necrotic patches that are not present in leiomyomas (3). Biopsy and histopathology are often the only definitive way to differentiate between the types of lesions; however, this was unfortunately not feasible in the case of this patient.

### Brief literature review

A recent systematic review found that most cases of IGF-II-mediated hypoglycaemia were associated with fibrous tumours (53.2%), followed by non-fibrous hepatic tumours (9%), haemangiopericytomas (8.4%), and mesotheliomas (4.7%) (4). To date, there are no confirmed reports of IGF-II-mediated bladder leiomyomas or leiomyosarcomas. However, a 2018 case report from the Japanese Urological Association described a bladder urothelial carcinoma producing IGF-II (5), and a 2001 study documented an IGF-II-secreting solitary fibrous tumour of the urinary bladder (6). Beyond these isolated cases, the literature linking bladder tumours to NICTH is sparse. Occasional reports of pelvic sarcomas associated with NICTH have been published (7), and IGF-II secretion has also been observed in uterine fibrous tumours (8). However, this was not relevant in the present case due to the patient’s history of bilateral salpingo-oophorectomy. In addition, a thoracic IGF-II-producing leiomyosarcoma was documented in 1988 (9). This report aims to broaden the spectrum of tumours implicated in NICTH and contribute to the limited body of the literature surrounding bladder leiomyomas or leiomyosarcomas.

### NICTH management

Initial management of NICTH involves oral or IV glucose and dietary measures to prevent further episodes of hypoglycaemia (10). Once a tumour has been identified, surgical resection remains the management of choice. Complete resection is considered a curative treatment with a minimal incidence of recurrence (10). Patients with frailty or significant progression of metastatic disease who are not surgical candidates have a more varied treatment course. Oral and IV diet and nutritional supplements have been trialled with mixed results (10).

Glucocorticoids are the most widely utilised medical treatment for NICTH. High-dose glucocorticoids demonstrate an immediate effect on symptoms and often resolve hypoglycaemia (10). Doses required can be as high as >200 mg of prednisolone (10). Glucocorticoids act by reducing IGF-II production and enhancing gluconeogenesis to bring up glucose levels (10). In addition, recombinant human growth hormone (rhGH) at high doses has shown success with the resolution of hypoglycaemia. Growth hormone (GH) suppresses peripheral glucose uptake, which subsequently increases levels of IGF-I, acid-labile subunit (ALS), and IGF-binding protein-3 (IGFBP-3) (10). These then promote production of IGF-II (10).

## Conclusion

This case highlights a rare presentation of NICTH likely secondary to a bladder tumour. Histopathological diagnosis was not confirmed due to patient preference; however, imaging and biochemistry suggested a diagnosis of either bladder leiomyoma or leiomyosarcoma. It demonstrates the importance of thorough investigation when faced with persistent hypoglycaemia, as it can frequently be due to an underlying paraneoplastic process. Surgical resection remains the mainstay of treatment for both bladder leiomyomas/leiomyosarcomas and non-islet cell tumours (10). Where resection is not possible, medical management can be attempted with diet, glucocorticoids, and recombinant human growth hormone (10). Clinicians should maintain a high index of suspicion for NICTH in similar clinical scenarios and be prepared to adopt a flexible, patient-centred approach to management, especially in older adults with limited physiological reserve.

## Declaration of interest

The authors declare that there is no conflict of interest that could be perceived as prejudicing the impartiality of the research reported.

## Funding

This research did not receive any specific grant from any funding agency in the public, commercial, or not-for-profit sector.

## Patient consent

Written informed consent for publication of their clinical details and clinical images was obtained from the patient’s next of kin. All patient information has been anonymised for confidentiality.

## Author contribution statement

IH, SS, and HK were all involved in the writing of this paper. HK was the consultant in charge of the patient’s care.

## References

[1] Vu A, Chik C & Kwong S. IGF-2-mediated hypoglycemia: a case series and review of the medical therapies for refractory hypoglycemia. Endocrinol Diabetes Metab Case Rep 2024 2024 23–89. (10.1530/EDM-23-0089) PMC10959053 38432069

[2] Khater N & Sakr G. Bladder leiomyoma: presentation, evaluation and treatment. Arab J Urol 2013 11 54–61. (10.1016/j.aju.2012.11.007) 26579246 PMC4442969

[3] Prihadi JC, Hengky A, Lionardi SK, et al. Characteristics and outcomes in bladder leiomyoma management: a systematic review of case reports and case series from the past 20 years. BMC Urol 2024 24 252. (10.1186/s12894-024-01624-3) 39538239 PMC11562614

[4] Ata F, Choudry HA, Khan AA, et al. A systematic review of literature on insulin-like growth factor-2-mediated hypoglycaemia in non-islet cell tumours. Endocrinol Diabetes Metab 2024 7 e00471. (10.1002/edm2.471) 38411039 PMC10897872

[5] Funada S, Kita Y, Okada Y, et al. Bladder urothelial carcinoma producing insulin-like growth factor II: a case report. Int J Urol Case Rep 2018 1 9–12. (10.1002/iju5.12018) PMC7292130 32743354

[6] Corti B, Carella R, Gabusi E, et al. Solitary fibrous tumour of the urinary bladder with expression of bcl-2, CD34, and insulin-like growth factor type II. Eur Urol 2001 39 484–488. (10.1159/000052490) 11306891

[7] Jannatalipour A, Panahi N, Pejman Sani M, et al. Non-islet cell tumor hypoglycemia (NICTH) associated with sarcoma: a case report. BMC Endocr Disord 2025 25 59. (10.1186/s12902-025-01885-5) 40045285 PMC11881327

[8] Wakami K, Tateyama H, Kawashima H, et al. Solitary fibrous tumor of the uterus producing high-molecular-weight insulin-like growth factor II and associated with hypoglycemia. Int J Gynecol Pathol 2005 24 79–84. (10.1097/01.pgp.0000148342.07759.a8) 15626921

[9] Daughaday WH, Emanuele MA, Brooks MH, et al. Synthesis and secretion of insulin-like growth factor II by a leiomyosarcoma with associated hypoglycemia. N Engl J Med 1988 319 1434–1440. (10.1056/NEJM198812013192202) 3185662

[10] Bodnar TW, Acevedo MJ & Pietropaolo M. Management of non-islet-cell tumor hypoglycemia: a clinical review. J Clin Endocrinol Metab 2013 99 713–722. (10.1210/jc.2013-3382) 24423303 PMC5393479

